# Seed Maturity and Extraction Solvent Shape the Antioxidant and In Vivo Anti-Inflammatory Activities of *Citrullus colocynthis* (L.) Schrad. Extracts: Implications for Dermo-Functional Use

**DOI:** 10.3390/plants15172597

**Published:** 2026-08-26

**Authors:** Belsem Marzouk, Assia Hamdi, Meher Refifà, Francesca Degola, Jamil Kraiem

**Affiliations:** 1 Laboratory of the Chemical, Pharmaceutical and Pharmacological Development of Drugs, Faculty of Pharmacy, University of Monastir, Monastir 5000, Tunisia; belsemmarzouk@yahoo.fr (B.M.); hamdiessia@gmail.com (A.H.); jamil.kraiem@fphm.u-monastir.tn (J.K.); 2 Department of Chemistry, Life Sciences and Environmental Sustainability, University of Parma, Parco delle Scienze 11/A, 43124 Parma, Italy; refifameher@gmail.com

**Keywords:** *Citrullus colocynthis*, medicinal plants, antioxidant activity, anti-inflammatory activity, xylene-induced ear oedema, seed maturity, phytotherapy

## Abstract

*Citrullus colocynthis* (L.) Schrad. (Cucurbitaceae) is a medicinal plant, renowned since antiquity, widely used in traditional medicine for the management of metabolic and inflammatory disorders: seed extracts particularly are reported for the management of rheumatoid arthritis, articular inflammation and skin wound recovery. Our aim was to comparatively evaluate the antioxidant and anti-inflammatory activities of aqueous and oily extracts obtained from mature and immature seeds, and to preliminarily assess their dermal tolerance and protective effects against induced inflammation and UV damage. Antioxidant activity was investigated in vitro using β-carotene bleaching and ferric reducing antioxidant power (FRAP) assays, whereas anti-inflammatory activity and anti-irritation effects were evaluated in vivo in a mouse model using the xylene-induced ear edema and the UV-induced skin irritation test. Oily extracts from both maturation stages showed marked inhibition of β-carotene oxidation, while the aqueous extract of immature seeds exhibited the highest ferric reducing capacity; on the other hand, fixed oils demonstrated a significantly greater inhibitory effect on oedema, suggesting a higher efficacy in modulating acute inflammatory responses. Results showed how *C. colocynthis* seed maturity and extraction solvent influence the bioactive potential of these ingredients for phytotherapeutic and dermo-functional applications, supporting a possible use against wrinkle formation and UV-induced skin alterations.

## 1. Introduction

Traditional medicine is founded on the use of medicinal plants for the prevention, diagnosis, and treatment of diseases. According to the World Health Organization (WHO), traditional medicine encompasses the totality of knowledge and practices—whether explicable or not—used to diagnose, prevent, or eliminate physical and mental disorders, relying exclusively on practical experience and empirically transmitted knowledge [1]. These practices vary considerably across countries and regions and are shaped by cultural, historical, and philosophical determinants.

Plants have consistently constituted an integral component of human life, serving both nutritional and therapeutic purposes. In recent decades, a renewed interest in natural products—often perceived as safer alternatives to synthetic compounds—along with increasing demand for organically derived substances, has stimulated scientific and commercial attention toward plant resources: in fact, epidemiological evidence indicates that regular consumption of plant-derived foods rich in phenolic compounds is associated with a decreased incidence of cardiovascular diseases, obesity, diabetes, and other chronic disorders [2]. Consequently, the exploitation of natural substances as sources of functional ingredients has emerged as a major research and development trend.

The medicinal use of plants dates back to prehistoric times and remains highly prevalent today. It is estimated that more than 80% of populations in developing countries rely predominantly on traditional medicine for primary healthcare needs. Furthermore, approximately 25% of pharmaceutical preparations worldwide are derived from plant sources [1]. In Tunisia, as in many developing countries, traditional pharmacopoeia remains widely practiced. Numerous herbal preparations are administered either as single remedies or in combination for the management of various pathologies. Within this framework and in the context of promoting the flora of Tunisia’s arid regions, particular attention has been directed toward a spontaneous species recognized for its medicinal properties.

*Citrullus colocynthis* (L.) Schrad., a perennial herbaceous plant belonging to the Cucurbitaceae family and commonly known as *colocynth* (or Ebucehilkarpuzu, Handhal, Pikrokolokunthia, Phidangourgia, Coloquintida, Visala, Kozalk, Haji Qawugh), it is one of the seven species belonging to the genus *Citrullus*; widely distributed across arid and semi-arid regions worldwide [3,4,5], in Tunisia it is locally referred to as *Handhal* or *Dellaa El Oued*, as it morphologically resembles a small, bitter watermelon also typical of this region.

The ethnopharmacological use of *C. colocynthis* has been renowned since antiquity for its therapeutic applications [6]; due to its rich phytochemical profile and diverse array of bioactive constituents including essential oils, glycosides, flavonoids, alkaloids, and fatty acids [7,8], it is particularly valued for its extensive nutraceutical, medicinal, and pharmaceutical applications [9]. Different organs of *C. colocynthis* are reported to be extensively utilized in traditional medicine for the treatment of various conditions [10], including diabetes, cancer, hypertension, inflammatory disorders, and certain urinary, gastrointestinal, and pulmonary infections: specific ethnobotanical records can be found in the USDA phytochemical and ethnobotanical database [11], a widely used reference that compiles traditional medicinal uses, phytochemical profiles, and pharmacological properties of plant species based on ethnobotanical records and scientific literature. Fruits and seeds have been recently reported to exert protective effects against metabolic and vascular disorders [12], and for the treatment of various conditions, including otalgia, dental pain, back pain, paralysis, mastitis, articular inflammation, and rheumatoid arthritis [13], but the traditional use of colocynth seeds has specifically been suggested to date back at a very early stage of human history: in fact, archaeobotanical and archaeogenomic evidence from Neolithic North Africa demonstrates that wild *Citrullus* species were exploited by humans as early as ~6000 BP, primarily for their seeds rather than pulp, as indicated by seed remains and consumption traces, supporting the early ethnobotanical use of bitter taxa such as *C. colocynthis* or closely related forms [14]. Interestingly, although the fruit pulp is the most cited medicinal part (especially as a purgative), seeds and seed oil are consistently reported in ethnobotanical sources as less toxic alternatives used in metabolic and topical applications, representing a safer and functionally distinct medicinal component compared to the highly toxic pulp.

Despite the growing interest in *C. colocynthis* as a medicinal plant, several aspects of seed extracts’ biological activity remain insufficiently explored. In particular, although previous studies have reported their pharmacological potential, in vivo evidence supporting its anti-inflammatory activity remains scarce, especially in relation to seed oil fractions, and to our knowledge no recent studies are available that examine the impact of seed maturity and extraction solvent on the antioxidant and anti-inflammatory properties of its seed extracts.

Skin aging is a multifactorial biological process driven by intrinsic mechanisms and exacerbated by extrinsic factors such as ultraviolet (UV) radiation, pollution, and oxidative stress. At the molecular level, excessive production of reactive oxygen species (ROS) contributes to lipid peroxidation, protein oxidation, DNA damage [15,16], and the activation of matrix-degrading enzymes, ultimately leading to collagen fragmentation, loss of elasticity, and wrinkle formation [17]. Consequently, the development of topical formulations enriched with natural antioxidants and bioactive lipids has attracted considerable scientific and industrial interest.

Given that oxidative stress and chronic low-grade inflammation are key contributors to cutaneous aging, the lipidic and antioxidant profile of *C. colocynthis* seed oil suggests a promising role as a functional ingredient in dermo-functional formulations. Its potential mechanisms of action may include free radical scavenging activity, modulation of inflammatory pathways, reinforcement of the skin barrier through essential fatty acids, and protection against collagen degradation, opening to the possible exploitation as photoprotective agents. However, the objectives of this study were to determine and characterize the chemical composition of seed oil and aqueous extracts, and to assess selected biological activities of these extracts, both in vivo and in vitro, including anti-radical capacity, anti-inflammatory activity and photoprotective potential.

## 2. Results

### 2.1. Physical Characterization of C. colocynthis Mature and Immature Seed Oils

A relative oil density of 0.983 g/cm^3^ was established at 20 °C for the fixed oils extracted from mature seeds, while 0.961 g/cm^3^ was determined for immature seed oils. This small variation suggests that seed maturation slightly influences the chemical composition of the oil, probably due to changes in fatty acid content during development, as we previously demonstrated (Table 1; [18]).

### 2.2. In Vitro Anti-Radical Activity Evaluation of C. colocynthis Seed Extracts

#### Inhibition of β-Carotene Bleaching

In this assay, the oxidation of linoleic acid oxidation generates various oxidation products (lipid hydroperoxides, conjugated dienes, and volatile by-products) that attack the chromophore of β-carotene, resulting in the bleaching of its characteristic color. However, the presence of antioxidants inhibits the bleaching of β-carotene induced by the oxidation products of linoleic acid.

**Figure 1:**
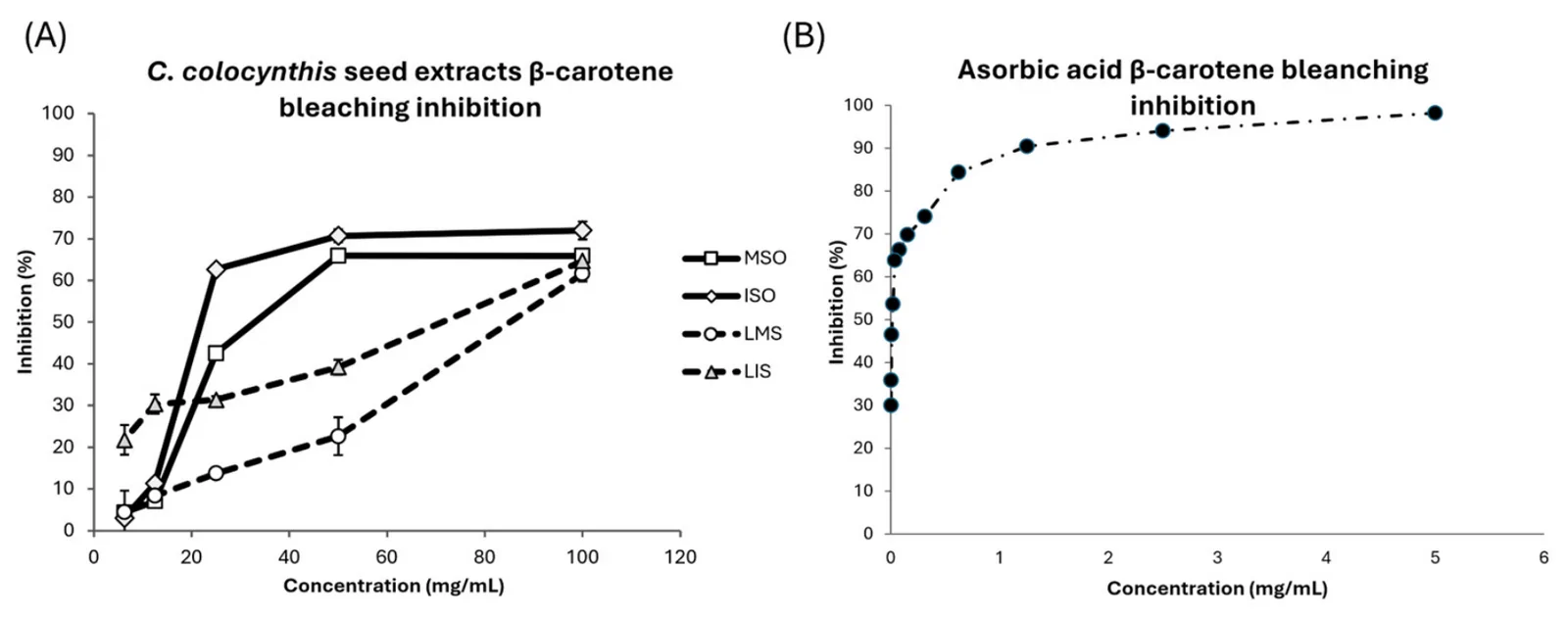
Results of β-carotene bleaching inhibition assay using different concentrations (mg/mL) of both fixed oils and lyophilized aqueous extracts from immature (ISO and LIS) or mature (MSO and LMS) *C. colocynthis* seeds (**A**). Ascorbic acid was used as a reference and reported (**B**). Data were reported ± SD.

As shown in Figure 1, the activity curves (expressed as the mean percentage inhibition) as a function of the concentration of the tested samples (expressed in mg/mL) exhibited two distinct phases: the first showed a rapid increase in anti-radical activity following an approximately linear trend at low concentrations. The second phase indicated that the effect continued to increase, but at a slower rate, eventually reaching a plateau for most of the parameters considered.

Immature seed oils (ISO) demonstrated the highest ability to inhibit radical formation from linoleic acid, with an inhibition percentage of 72%, followed by mature seed oils (65.89%). Freeze-dried extracts of both mature and immature seeds showed lower antioxidant activity compared to the oil extracts, with inhibition values of 61.60% and 64.66%, respectively. Nevertheless, all extracts exhibited significant inhibitory effects on the oxidation of the β-carotene/linoleic acid system, comparable to ascorbic acid at a concentration of 5 mg/mL, which showed an activity of 98.18%. Therefore, ISO can be considered to possess the highest antioxidant capacity.

### 2.3. Potassium Ferricyanide Reducing Power Assay

**Figure 2:**
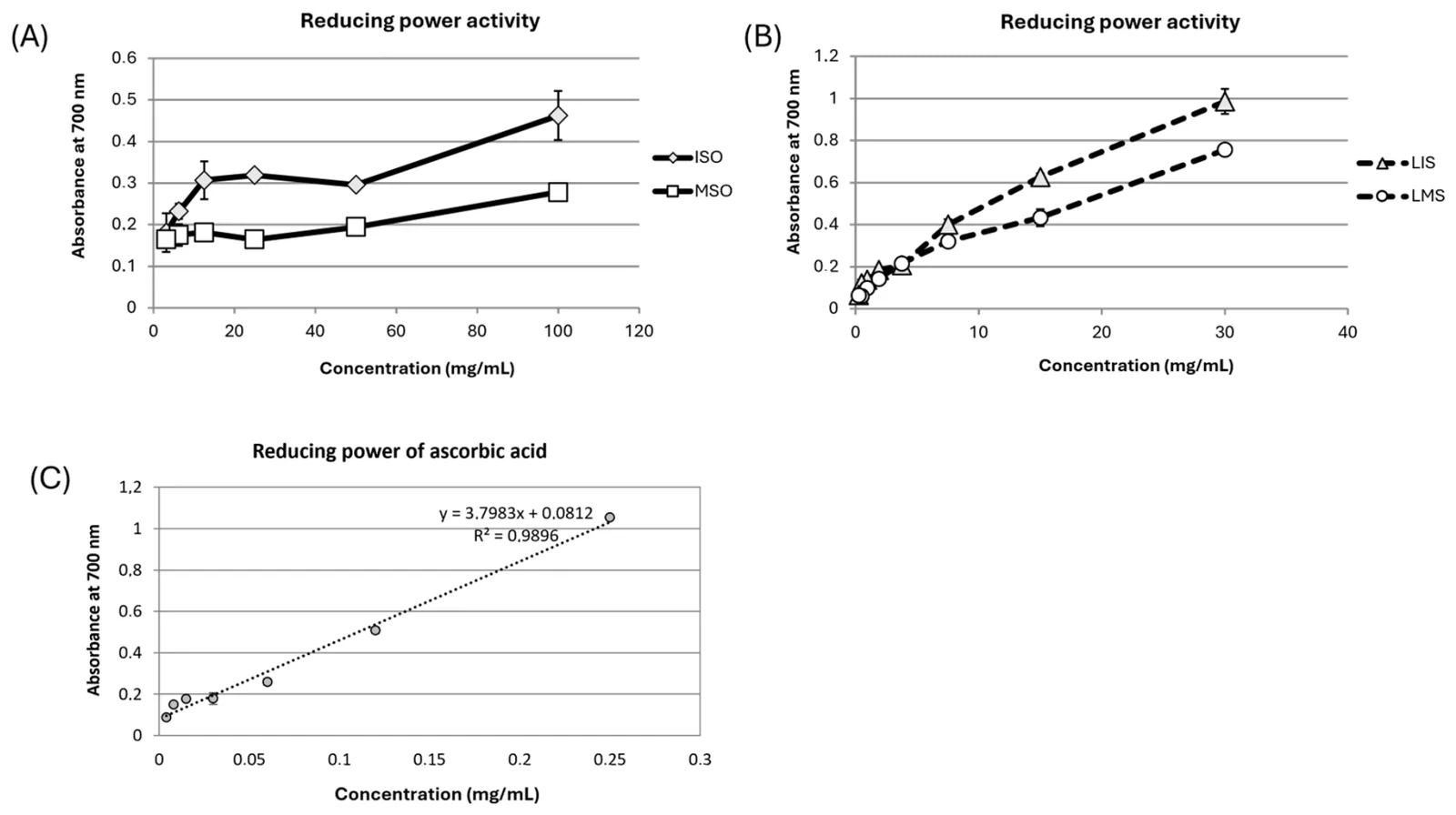
Reducing power activity of fixed oils (**A**) and lyophilized aqueous extracts (**B**) from immature (ISO; LIS) and mature (MSO; LMS) *C. colocynthis* seeds. Ascorbic acid was used as a reference and reported (**C**). Data were reported ± SD.

As a complementary test, the potassium ferricyanide reducing power assay method was used. This test, based on the ability of polyphenols to reduce ferric iron (Fe^3+^) to ferrous iron (Fe^2+^) [19] is a rapid, reproducible, and easy-to-perform analysis. Results of the reducing activity of both aqueous and fixed oils are shown in Figure 2.

In contrast to the β-carotene/linoleic acid assay, the ferricyanide reducing power assay yielded different results: in fact, at a concentration of 100 mg/mL, the reducing power of immature and mature seed fixed oils (ISO and MSO) was 0.462 and 0.278, respectively (Figure 2A), values that are markedly lower than that obtained for ascorbic acid at 1 mg/mL (2.83; Figure 2C). On the other hand, the lyophilized aqueous extracts of both immature and mature seeds exhibited higher reducing capacity at 30 mg/mL (0.986 and 0.756, respectively; Figure 2B).

The free radical scavenging activity of the extracts was also evaluated using both the β-carotene bleaching assay (coupled with linoleic acid) and the ferricyanide reducing power assay, and expressed as IC_50_, defined as the concentration of extract required to reduce 50% of the free radicals present in the solution. Lower IC_50_ values indicate greater free radical scavenging activity. For comparison, ascorbic acid was used as a reference standard. Although all *C. colocynthis* seed extracts exhibited lower antioxidant activity than the standard (Table 2), they still demonstrated strong scavenging capacity, as evidenced by their relatively low IC_50_ values (10.78 and 18.24, respectively). Two-way ANOVA revealed a significant interaction between extraction type and seed maturation for both antioxidant assays, indicating that the effect of seed maturity on antioxidant activity depended on the extraction solvent.

### 2.4. In Vivo Anti-Inflammatory Activity

The acute anti-inflammatory activity of extracts prepared from mature and immature *C. colocynthis* seeds was investigated using the xylene-induced ear edema model in mice. The results showed a dose-dependent, anti-inflammatory effect: according to Table 3, mature seed oil showed the highest ability to significantly suppress ear edema with an inhibition percentage of 95.56%, followed by seed oil from immature seeds with an inhibition rate of 82.24%. Lyophilized seed extracts had a lower anti-oedema effect when compared to fixed oil extracts. Also, at the concentration of 5 mg/kg, mature seed fixed oil determined a more vigorous anti-inflammatory effect (95.5%) when compared to that of the reference (Dexamethasone). However, the respective values of the percentages of inhibition of edema under the effect of the other extracts (immature seed fixed oils and lyophilized seed extracts) always remain slightly lower than those recorded with the reference product.

### 2.5. In Vivo Preliminary Evaluation of Photoprotective Potential Against UV-Induced Irritation

A simple water-in-oil (W/O) cream was selected as the vehicle for in vivo testing to ensure a stable and biocompatible delivery of the plant extracts while minimizing interference from formulation components. Such basic emulsions are widely used in topical studies as inert carriers, allowing the evaluation of the intrinsic anti-irritant activity of the tested compounds under controlled conditions.

**Figure 3:**
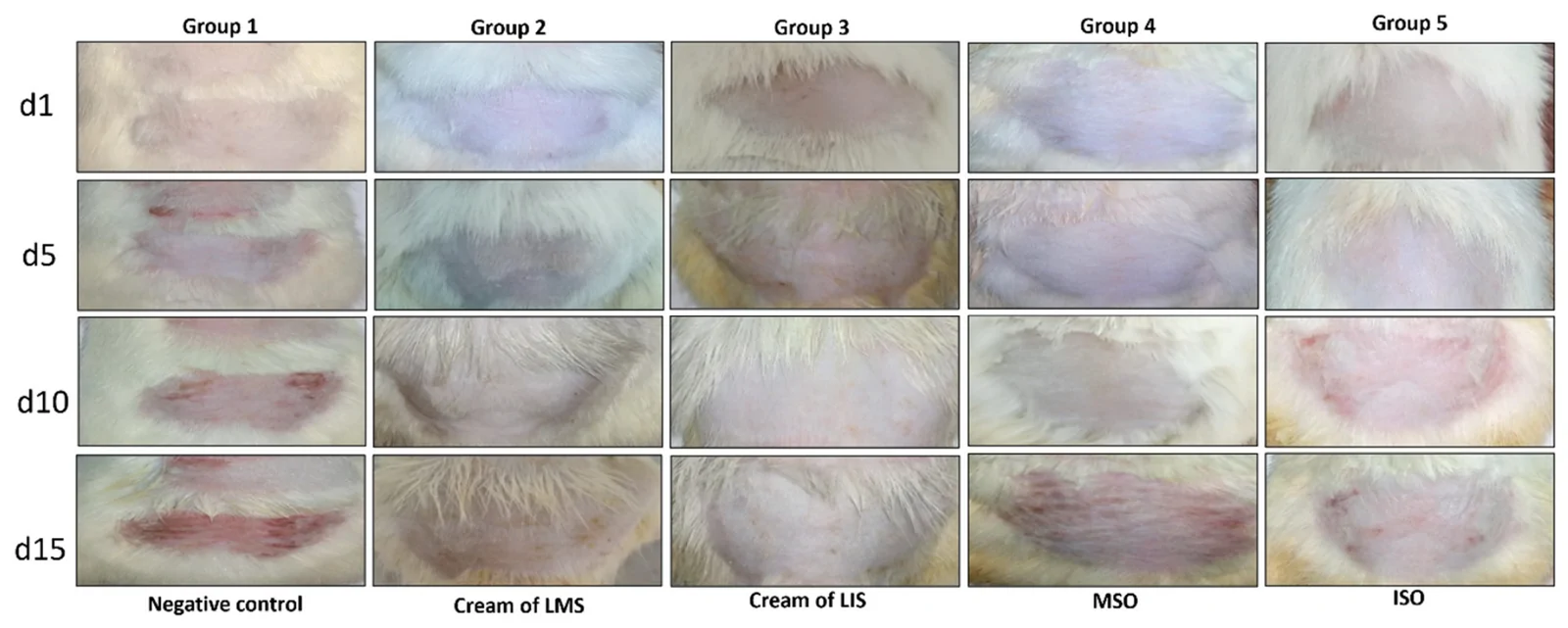
In vivo skin irritation test to evaluate the photoprotective potential of *C. colocynthis* seed extracts. Skin appearance of rats observed at 1, 5, 10 and 15 days of UV exposure.

UV-induced skin irritation tests showed that the simple application of blank cream resulted in a visible development of erythema in 5 days of treatment (Figure 3); on the contrary, no erythema or edema were observed in the dorsal coastal region of rats belonging to groups 2, 3 and 4, not even after 10 days of UV exposure. Amongst fixed oil formulations, ISO resulted the most effective in delaying the effects of UV-induced skin, while the two formulations with lyophilized aqueous seed extracts were comparable.

## 3. Discussion

The chemical composition of *C. colocynthis* fruit and seeds is known to be affected by the environmental conditions during maturation, as the plant is highly sensitive to the environment [20]. In some regions, fruits are harvested between May and October, with the ripening process during the hot summer affecting the final oil, protein, and fat contents. Additionally, despite increasing interest in this medicinal plant, the impact of seed maturity and extraction solvent on its biological activity has not been systematically investigated so far.

Here, the difference observed in the oil density between immature and mature seeds reflected a change in the chemical composition of seed oil during the maturation process, a common phenomenon in oilseed species and already documented in *C. colocynthis* [18]. Oil from mature seeds has been found to be dominated by linoleic acid (ranging from 50% to over 70% in different studies), followed by oleic acid (10–25%), palmitic acid (8–12%), and stearic acid (5–8%), with a degree of unsaturation higher than 75% (typical of semi-drying oils) [18,21,22]. Hence, the density of oil depends primarily on the nature and proportion of its fatty acids: the denser oil obtained from mature seeds indicated a higher content of saturated fatty acids or heavy compounds (such as complex triglycerides, sterols, pigments, etc.), which is characteristic of more stable and less volatile oils. Conversely, the lower density observed for immature seed oil seemed to indicate a higher proportion of unsaturated fatty acids or light substances, often associated with less stable but more fluid oils. Our observations confirmed a significant change in the physicochemical characteristics due to seed maturation, which should be taken into account when considering *C. colocynthis* seeds as plant material for pharmacological applications.

Based on the results obtained from the in vitro evaluation of antioxidant activity, we concluded that the lyophilized aqueous extract from immature seeds (LIS) possessed the most effective free radicals inactivation potential, which we speculate can be attributed to the presence of flavonoids, which are the major constituent of immature seeds of *C. colocynthis* [23,24] and are acknowledged as powerful antioxidants, playing the role of inhibitors for different oxidative species (such as OH·, diphenylpicrylhydroxide radical, superoxide) and also preventing lipid peroxidation [19]. Findings were consistent with what was previously described [25]: in fact, fixed oils from both *C. colocynthis* immature and mature seeds, which contained fewer chemical components than the aqueous extracts, exhibited much less significant anti-radical activity. Under these conditions, it can be assumed that there is synergy between the numerous chemical classes contained in the aqueous extracts. On the other hand, the absence of phenolic compounds in seed fixed oils is likely responsible for the less effective anti-radical effect observed.

An evaluation of the in vivo anti-inflammatory potential of *C. colocynthis* seed extracts was preliminary to further investigations about the photoprotective effects of extracts themselves, as inflammation represents the primary response to UV damage at the skin level: in fact, exposure to UV radiation (and especially UVB) is known to trigger redness, swelling, and the release of pro-inflammatory mediators, all processes that are central to photodamage [26,27]. This makes anti-inflammatory activity a relevant early indicator of potential photoprotective benefit. Xylene-induced ear edema in mice is one of the appropriate models for evaluating anti-inflammatory activity, as xylene is a phlogogenic agent that causes fluid accumulation, which subsequently leads to the formation of edema, characteristic of acute inflammation [28]. The molecular and cellular mechanism of xylene-induced inflammation involves capsaicin-sensitive sensory neurons, which, upon stimulation, release substance P, that acts as a neurotransmitter or neuromodulator in several physiological processes [28]. In our study, all the extracts tested showed a significant anti-edema activity, but the strongest effect was observed with fixed oils from both mature and immature seeds: this might be explained by the presence of high amounts of linoleic acid associated with behenic acid [18], since these two fatty acids are endowed with anti-inflammatory activity reported in several studies [29,30,31]. Linoleic acid, in particular, that acts as a modulator of inflammatory mediators such as immunoglobulins, cytokines and prostaglandins, is capable of controlling the mechanism of inflammation by stimulating the secretion of soluble factors [32,33].

The anti-inflammatory efficacy demonstrated by *C. colocynthis* extracts was found to correlate with significant efficacy against UV-induced skin irritation, as all the tested formulations exhibited skin-protective properties and contributed to reducing UV-induced erythema. Antioxidant power is a central point in combating oxidative stress, the main factor in skin aging. By neutralizing reactive oxygen species (ROS), they protect collagen and elastin fibers against enzymatic degradation induced by matrix metalloproteinases, thus helping to preserve skin elasticity and firmness [34]. Furthermore, several flavonoids such as quercetin and apigenin have demonstrated the ability to stimulate collagen synthesis and modulate cell signaling involved in tissue regeneration, thus promoting the wound healing process. They also exhibit anti-inflammatory and antimicrobial activity, limiting leukocyte infiltration and microbial colonization of wounds, essential conditions for rapid and high-quality healing [35]. Polyphenols, and especially flavonoids, represent promising candidates for preventing skin aging and improving tissue repair. In vitro and in vivo studies show that administering flavonoids both topically and systemically offers numerous benefits, such as slowing skin cell senescence and preventing signs of aging [36]. Fatty acids are also widely studied for their role in skin healing: linoleic acid in particular not only promotes faster skin regeneration by accelerating healing and reducing hypertrophic scars, but also improves the aesthetic appearance [37]. Accordingly, the predominance of unsaturated fatty acids in *C. colocynthis* seed extracts, especially linoleic acid, may represent an additional factor underlying their antioxidant and anti-inflammatory properties and their potential benefits for skin protection in dermo-functional formulations.

## 4. Materials and Methods

### 4.1. Plant Material

At least 10 fruits of *Citrullus colocynthis* adult plants were collected from each of five independent populations, located in the municipality of Sidi Makhlouf (Medenine, Tunisia; 33°33′ N, 10°27′ W); the identification at species level was obtained according to the flora of Tunisia [38]. Seeds were obtained from both mature and immature fruits and processed separately; the maturation stage was assessed using visual inspection: according to the species phenology, fruits with bright green skin featuring distinct irregular lighter green or white vertical stripes and a fleshy interior with high moisture content were classified as immature, while fruits turned brownish, yellowish, or light leather-colored, lightweight and hard-textured were classified as fully ripened. Seed maturity was also established according to morphological characteristics: white to cream-colored seeds with small dimensions, soft texture and high water content were classified as immature, while light brown, maximum-sized seeds with a hard, dry texture and low water content were classified as mature.

### 4.2. Seed Fixed Oils Extraction and Characterization

Immature and mature seeds were dried and powdered with a tissue blender. Different solvents, in ascending polarity (petroleum ether, chloroform, ethyl acetate, and methanol), were used for Soxhlet extraction to fractionate the soluble compounds from the plant material. The extraction was performed on dried powder (100 g) placed inside a thimble made of thick filter paper loaded into the main chamber of the Soxhlet extractor. The total extraction time was 6 h for each solvent, continuously refluxing over the sample at a temperature not exceeding the boiling point. The resulting extracts were evaporated under reduced pressure to obtain the crude extracts. The organic solvents used were 99% pure. All the chemicals were purchased from Sigma-Aldrich Corp. (St. Louis, MO, USA). Only petroleum ether extracts were used for the experiments. Extraction yield was 8.96% and 7.81% for immature and mature seeds, respectively. The chemical composition of fixed oils was determined by analyzing the Fatty Acid Methyl Esters (FAMEs) by GC-FID, according to Marzouk et al. [18]. Briefly: FAMEs were prepared by shaking solutions of fixed oils in hexane (0.1/mL) with 0.1 mL BF3-MeOH (14%); the reaction was incubated for 30 min at 70 °C, then stopped by adding 0.5 mL ultrapure water. The fatty acid methyl esters were then analyzed on a Hewlett-Packard (HP 5890, series II) gas chromatograph (Hewlett-Packard Ca., Palo Alto, CA, USA) containing a split/split less injector and a flame ionization detector (FID) linked to an HP Chemstation integrator. A fused-silica capillary column HP-Innovax (30 m × 0.25 mm × 0.25 μm) was used with nitrogen gas at a flow rate of 1 mL/min, flame ionization detection temperature 280 °C, injector (split) temperature 250 °C with an injection volume of 1 μL and a split ratio of 1:50. The oven temperature was programmed from 180 to 250 °C. FAMEs were identified by comparing their relative and absolute retention times to those of TFA standards, and results are expressed as a percentage of total area.

### 4.3. Fixed Oils Density Determination

The relative density of the fixed oils (D20), defined as the ratio of the mass of a given volume of oil at 20 °C to the mass of an equal volume of distilled water at 20 °C, was determined using a calibrated pycnometer. The empty pycnometer was weighed (m0), filled with distilled water previously boiled and cooled, thermostated at 20 °C, and weighed again (m1). The same procedure was repeated using the oil samples to obtain m2. The relative density was calculated according to the following equation: D20 = (m2 − m0)/(m1 − m0), where m0 is the mass of the empty pycnometer, m1 is the mass of the pycnometer filled with distilled water, and m2 is the mass of the pycnometer filled with oil [39].

### 4.4. Seed Aqueous Extracts Lyophilization and Cream Preparation

A total of 100 g of either immature or mature seeds were ground with a mixer and added to 500 mL of distilled water. To ensure efficient extraction of the bioactive compounds, the mixture was allowed to be refluxed for 30 min, after which the solution was allowed to cool for four hours at 4 °C. The mixture was then filtered using filter paper (Whatman no. 1) under a vacuum water pump. The filtrates obtained were lyophilized. Extraction yield was 2.94% and 2.21% for immature and mature seeds, respectively. A phytochemical screening for the presence of major antioxidant bioactives (alkaloids, coumarins, flavonoids, anthraquinones, cardiac glycosides, iridoids, saponins and tannins) was conducted and already reported in Marzouk et al. [25].

From the obtained powders, creams were prepared according to Lassoued et al. [40]; briefly, oil/water emulsions were prepared as follows: both oil (15% olive oil, 5.5% cetyl alcohol, 2.75% sorbitan monostearate) and aqueous phase (2.25% POE (20) sorbitan monostearate, 5% glycerin, 5% lyophilized aqueous extract, water up to 64%) were heated separately to 75 °C under gentle stirring (250 rpm) before mixing for 10 minutes. The emulsion was passed through a homogenizer and kept under stirring until cooling to room temperature. Finally, Sepicide^®^ HB (La Garenne Colombes, France) was added as a preservative when the temperature reached 40 °C, and the cream was then stirred for five additional minutes. The pH value was assessed for both blank (without extract) and test (added with lyophilized water extracts) creams, to check the compatibility with the physiological skin range (typically ranging from 5 to 7). All pH values measured were between 6.5 (blank) and 5.5 (test creams).

### 4.5. In Vitro Anti-Radical Activity Evaluation

#### 4.5.1. β-Carotene Bleaching Assay Coupled with Linoleic Acid Oxidation

In a ground-necked flask, 1.5 mg of β-carotene was added with 2.5 mL of chloroform. The chloroform solution of β-carotene was mixed with 20 µL of linoleic acid and 200 µL of Tween (20%). The mixture was evaporated using a rotary evaporator at a temperature of 35 °C. After evaporation, with vigorous stirring, a volume of 50 mL of distilled water was added. 200 µL of the different concentrations of extracts (aqueous and oils) of ripe and immature gourd seeds were each mixed with 800 µL of the β-carotene/linoleic acid emulsion solution. The absorbance of different concentrations was measured spectrophotometrically at 470 nm immediately (T = 0) and after 2 h incubation at 50 °C (T = 120 min). The concentration of the extract providing 0.5 absorbance (IC_50_) was calculated from the graph. Ascorbic acid was used as a standard. The percentage inhibition is calculated using the following formula: % inhibition = [1 − (E0 – E120)/(C0 – C120)] × 100, where: E0 = the solution containing the extract before incubation; E120 = Absorbance of the solution containing the extract after 120 min incubation at 50 °C; C0 = Absorbance of the control (without extract) before incubation; C120 = Absorbance of the control (without extract) after 120 min incubation at 50 °C [41].

#### 4.5.2. Reducing Antioxidant Power Assay

The potassium ferricyanide reducing power assay was used. A total of 200 µL of each extract, at different concentrations (up to 100 mg/mL), was mixed with 500 µL of a 0.2 M phosphate buffer solution (pH 6.6) and 500 µL of a 1% potassium ferricyanide solution. The reaction medium was incubated in a water bath at 50 °C for 20 min, then 500 µL of 10% TCA (trichloroacetic acid) was added to block the reaction. The tubes were centrifuged at 3000 rpm for 10 min, then 500 µL of supernatant was mixed with 500 µL of distilled water and 100 µL of a 0.1% aqueous FeCl_3_ solution. Finally, the absorbance was read at 700 nm using a spectrophotometer. The concentration of the extract providing 0.5 absorbance (IC_50_) was calculated from the graph. Ascorbic acid was used as a standard. The increase in absorbance is proportional to the resulting reducing power [42]. The percentage of iron reducing power is calculated using the following reaction: [(Abs 700 Control − Abs 700 Extract)/Abs 700 Control] × 100.

### 4.6. In Vivo Evaluation of Anti-Inflammatory Activity

Anti-inflammatory activity was assessed using the xylene-induced ear edema model according to Kou et al. [43] with slight modifications. Both oil and lyophilized seed extracts were properly diluted in an injectable solution (0.25 mL ethanol, 0.25 mL Tween30^®^ and 2 mL water). Swiss albino male rats (25–30 days old) were randomly divided into groups, each containing 5 animals per dose: negative control (nothing received), positive control (Dexamethasone), immature seeds oil treatment (ISO), mature seeds oil treatment (MSO), lyophilized water immature seeds extract treatment (LIS) and lyophilized water mature seeds extract treatment (LMS). Treatments were applied subcutaneously, at increasing doses (from 0.25 to 5.00 mg/kg), on both ears; then, 30 min after administration of either treatments or Dexamethasone (5.00 mg/kg), animals were induced with topical xylene for inflammatory response: 30 µL of the phlogogenic agent were applied to the inner and outer surfaces of the right ear of each mouse, while left ear was kept as inner control. Three hours after topical xylene administration, ear thickness was measured using a digital caliper. For each animal, treatments were applied to one ear, while the contralateral ear was used as the internal control; the percentage inhibition was therefore calculated from paired measurements obtained from the two ears of the same animal, then the mean of differences between the right and left ears was determined for each group, according to the following formula: Inhibition percentage (%) = [1 − (∆e (test)/∆e (negative control))] × 100, where ∆e (test) = Average difference in thickness between the two ears in the treated group, and ∆e (negative control) = Average difference in thickness between the two ears in the untreated group.

### 4.7. In Vivo UV-Induced Skin Irritation Test

In order to assess the potential photoprotective potential of *C. colocynthis* seed extracts, which could support their use as an ingredient for pharmaceutical skin anti-aging formulations, an in vivo, UV-induced skin irritation test was performed. Hair from the dorsal area (about 2 cm × 3 cm) of rats was carefully removed with an electric shaver, then animals were divided into five groups (*n*-5): (i) Group 1 (negative control group), treated with white cream only; (ii) Group 2, that underwent daily application of a cream prepared from mature seeds; (iii) Group 3, that underwent daily application of a cream prepared from immature seeds; (iv) Group 4, that underwent daily application of the fixed oil from the mature seeds; (v) Group 5, that underwent daily application of fixed oil from immature seeds. Creams or oils were applied in a thick layer on the shaved and depilated part of the rat, which were then irradiated using a UV lamp (Camag Muttenz schweiz 29200, Camag Chemie-Erzeugnisse und Adsorptionstechnik AG, Muttenz, Switzerland. 220 V, 50 Hz): all rats were anesthetized and then subjected to daily exposure to irradiation for 20 min at 366 nm (UVA) up to 15 days. The shaved and waxed area was placed 10 cm from the lamp, while the rest of the body was protected by an opaque cloth. Photographs were taken daily, just before each irradiation session, to monitor the appearance of wrinkles and/or erythema.

### 4.8. Statistical Analysis

Statistical analyses were performed using two-way ANOVA with extraction type and seed maturation as fixed factors, and their interaction term was evaluated. Normality (Shapiro–Wilk test) and homogeneity of variance (Levene’s test) were assessed prior to ANOVA. Post-hoc comparisons were conducted using Duncan’s multiple range test. The positive control (ascorbic acid) was analyzed separately using one-way ANOVA. Statistical analyses were conducted using IBM SPSS Statistics v30, and differences were considered significant at *p* < 0.05.

## 5. Conclusions

The present study demonstrates that *C. colocynthis* seed extracts possess significant antioxidant and anti-inflammatory activities, with marked differences depending on the extraction solvent and the stage of seed maturation. Oily extracts exhibited superior radical-scavenging capacity -in the β-carotene bleaching assay- compared to aqueous extracts and showed a stronger inhibitory effect in the xylene-induced ear edema model, indicating a greater efficacy in modulating acute inflammatory responses. In contrast, the aqueous extract of immature seeds displayed the highest ferric reducing power.

Our findings, which are consistent with the traditional use of *C. colocynthis* in the management of inflammatory conditions, also highlight the importance of both extraction methods and plant developmental stage in determining the biological activity of plant-derived products. Preliminary results from the in vivo tests suggest that the tested formulations are compatible with topical application, although further studies are required to confirm their long-term safety and efficacy. Additionally, the potential protective effects against skin aging and UV-induced alterations remain to be validated through dedicated photobiological and mechanistic investigations.

Taken together, these findings not only support the potential of *C. colocynthis* seeds as a source of bioactive compounds for phytotherapeutic applications, providing a basis for further investigations aimed at elucidating their mechanisms of action and clinical relevance, but also contributes to the valorization of Tunisia’s native flora, particularly species adapted to arid and semi-arid ecosystems; in fact, since *C. colocynthis* ia a resilient genetic resource with significant yet underexploited biotechnological potential, the scientific characterization and functional assessment of its seed oil is expected to promote the sustainable use of local plant biodiversity, encourage the development of value-added phytoproducts, and support regional bioeconomy strategies. In this context, the exploration of indigenous species for dermocosmetic applications aligns with current efforts to integrate traditional knowledge, biodiversity conservation, and innovation in natural product research.

## Figures and Tables

**Table 1:** Fatty acid composition of fixed oils from *C. colocynthis* immature (ISO) and mature (MSO) seeds. Data are reported as a percentage of total fatty acid content.

RT (min)	Fatty Acids	FA Abbreviation	Composition (%)	
			ISO	MSO
3.7	Myristic acid	C14:0	0.06	0.07
4.3	Myristoleic acid	C14:1	0.04	0.05
5.7	Palmitic acid	C16:0	7.50	6.31
6.0	Palmitoleic acid	C16:1(n7)	0.16	0.46
8.6	Stearic acid	C18:0	0.13	4.84
9.0	Oleic acid	C18:1(n9)	26.94	20.94
9.8	Linoleic acid	C18:2(n6)	61.20	59.05
10.7	α-Linoleic acid	C18:3(n3)	0.63	1.09
12.5	Arachidic acid	C20:0	0.55	0.49
13.6	Gadoleic acid	C20:1(n9)	0.04	0.01
14.6	Eicosadienoic acid	C20:2(n6)	0.01	0.16
15.5	Heneicosylic acid	C21:0	0.43	1.83
16.5	Behenic acid	C22:0	2.31	5.10

**Table 2:** Free radical scavenging potential, calculated as IC_50_ (mg/mL), of *C. colocynthis* immature (ISO) and mature (MSO) seed oil extracts, lyophilized mature (LMS) and immature (LIS) seed extracts. Ascorbic acid was used as a reference. Data were reported ± SD. Different letters indicate statistically significant differences at *p* ≤ 0.05.

	ISO	MSO	LMS	LIS	Ascorbic Acid
**β-carotene**	18.242 ± 0.722 ^b^	21.870 ± 0.198 ^bc^	32.611 ± 2.554 ^d^	65.179 ± 2.183 ^e^	0.015 ± 0.002 ^a^
**Ferricyanide**	99.005 ± 0.000 ^e^	>100 mg	18.002 ± 1.513 ^c^	10.789 ± 0.698 ^b^	0.090 ± 0.007 ^a^

**Table 3:** Potential of oedema inhibition of *C. colocynthis* immature (ISO) and mature (MSO) seed oil extracts, and lyophilized mature (LMS) and immature (LIS) seed extracts. Dexamethasone was used as a reference. Different letters indicate statistically significant differences at *p* ≤ 0.05.

Extract	Dose (mg/kg)	Oedema Inhibition (%)
ISO	0.25	26.036 ± 0.083 ^c^
	0.50	55.621 ± 0.050 ^f^
	1.00	57.840 ± 0.048 ^g^
	2.50	58.580 ± 0.047 ^g^
	5.00	82.249 ± 0.020 ^i^
MSO	0.50	28.994 ± 0.080 ^cd^
	1.00	35.651 ± 0.073 ^d^
	2.50	54.142 ± 0.052 ^f^
	5.00	95.562 ± 0.005 ^j^
LMS	0.50	17.900 ± 0.093 ^b^
	1.00	22.338 ± 0.088 ^bc^
	2.50	51.184 ± 0.055 ^f^
	5.00	71.154 ± 0.033 ^h^
LIS	0.50	8.284 ± 0.103 ^a^
	1.00	33.432 ± 0.075 ^d^
	2.50	40.829 ± 0.067 ^e^
	5.00	80.769 ± 0.022 ^i^
Dexamethasone	5.00	68.94 ± 9.31 ^h^

## Data Availability

The original contributions presented in this study are included in the article. Further inquiries can be directed at the corresponding author.
